# Dual-chamber pacing with closed-loop stimulation in a sleep syncope case

**DOI:** 10.1016/j.hrcr.2024.06.015

**Published:** 2024-06-22

**Authors:** Junji Morita, Yuhei Kasai, Takayuki Kitai, Tsutomu Fujita, Yusuke Kondo

**Affiliations:** ∗ Department of Cardiology, Sapporo Cardiovascular Clinic, Sapporo, Japan; † Department of Cardiovascular Medicine, Chiba University Graduate School of Medicine, Chiba, Japan

**Keywords:** Closed-loop stimulation, Pacemaker, Sleep syncope, syncope, Vasovagal reflex syncope

**Key Teaching Points**

• Sleep syncope is considered a subtype of vasovagal reflex syncope (VVS). Closed-loop stimulation (CLS) has improved VVS-related sleep syncope.
• This case report revealed the detailed pacing activity state in CLS treatment for VVS.
• CLS resulted in heart rate stability. Cycle of elevated heart rate (HR) followed by activated CLS and dropped HR was not observed.
• The Holter monitoring showed cyclically atrial pacing following HR elevation during the night. It was suggested that the CLS is functioning appropriately in response to the sympathetic nervous system activation during sleep, which is a mechanism of sleep syncope.

## Introduction

Sleep syncope is classified as a subtype of vasovagal reflex syncope (VVS).^1^ Closed-loop stimulation (CLS) is helpful for managing VVS.^2^^,^^3^ In this report, we present a case in which CLS was effective for sleep syncope.

## Case report

**Figure 1:**
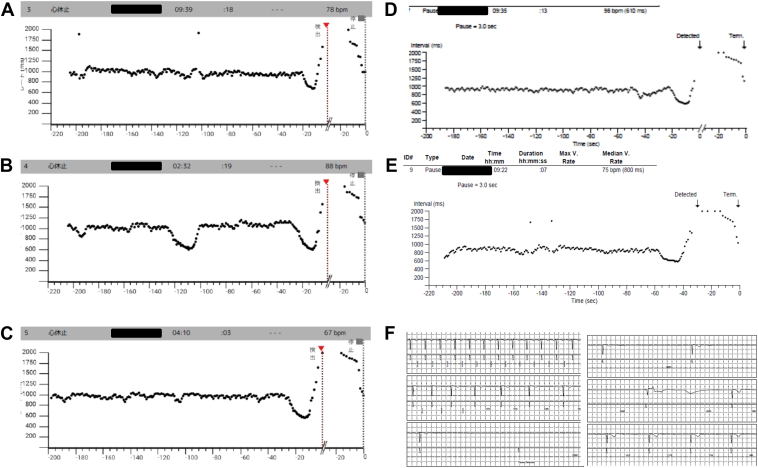
Five episodes of asystole were recorded on an insertable cardiac monitor over 2 months. In all 5 episodes, asystole is observed following a trend of sinus tachycardia. **A, B, C:** Recordings of episodes observed during sleep. **D, E:** Recordings of episodes observed throughout the day. **F:** Detailed electrogram of episode shown in panel E.

**Figure 2:**
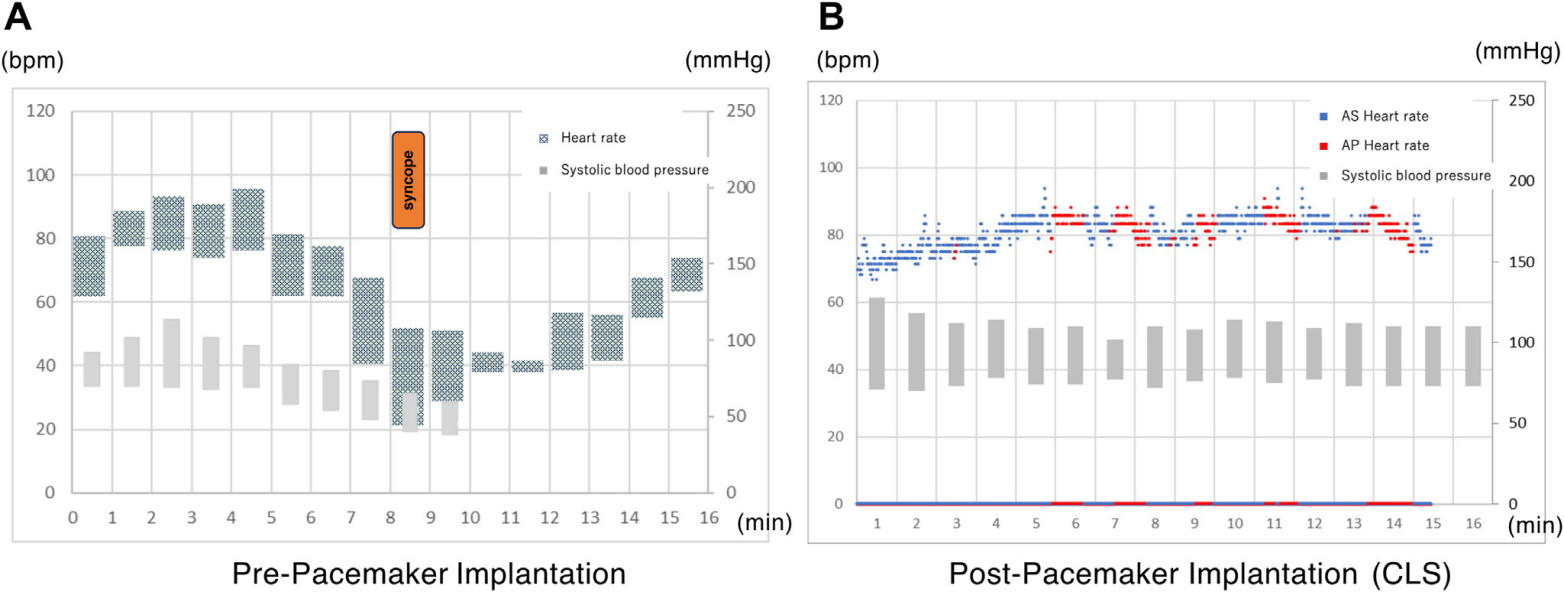
Systolic and diastolic blood pressure are measured per minute. The heart rate is monitored continuously, showing the minimum and maximum heart rates (HR) per minute before pacemaker implantation. Closed-loop stimulation (CLS) is measured at every beat to distinguish between atrial sensing and atrial pacing after pacemaker implantation. **A:** Syncope occurs before pacemaker implantation. **B:** As a result of atrial pacing induced by the activation of CLS, a stabilization in HR is observed without any decrease.

A 53-year-old woman with no specific medical history visited another hospital because of recurrent syncope following a prolonged standing time. As epilepsy was suspected, repeated electroencephalographic examinations were performed; however, no abnormalities were found. After insertable cardiac monitor (ICM) implantation at another hospital, the patient was referred to our hospital for pacemaker implantation because of repeated episodes of asystole recorded on the ICM. Five episodes of asystole (lasting >3 seconds) were recorded over 2 months. In all 5 episodes, asystole was observed following a trend of sinus tachycardia (Figure 1). Of the 5 episodes, in 2 episodes of asystole the patient fainted after prolonged sitting (Figure 1D and 1E). The remaining 3 episodes occurred during sleep (Figure 1A, 1B, and 1C). Details of the episodes of asystole during sleep were as follows: the first time, the patient awoke with awareness of urinary incontinence; the second time, the asystole was asymptomatic and the patient was unaware of it; and the third time, she awoke feeling nauseated, then shifted to a resting supine position, resulting in supine syncope. We performed head-up tilt (HUT) testing and an electrophysiological study (EPS) on admission. We continuously monitored the heart rate (HR) during the HUT, observing the minimum and maximum HR for each minute. The HUT revealed a cardioinhibitory response because syncope occurred with a decreased HR of 24 beats per minute and blood pressure at 8 minutes after the start without drug loading (Figure 2A). The sinus node recovery time in the EPS was normal, at 1040 ms. No sleep study was conducted owing to the lack of typical sleep apnea symptoms, such as loud snoring, excessive daytime sleepiness, morning headaches, and dizziness upon waking. Based on a comprehensive evaluation of the patient’s condition at the time of syncope, ICM recordings, HUT test, and EPS, the patient was diagnosed with VVS. We implanted a dual-chamber pacemaker with CLS (Biotronik, Berlin, Germany) considering the recurrent syncope and prolonged asystole. The pacemaker settings were DDD-CLS 45/120, CLS rate 110, and high CLS. HUT was performed 5 days after implantation to validate the effect of CLS. A 12-lead electrocardiogram (ECG) was performed during the HUT examination to check for pacing spikes. Furthermore, CLS was evaluated at every beat to determine whether there was a spike in the P wave, to distinguish between atrial sensing and atrial pacing.

**Figure 3:**
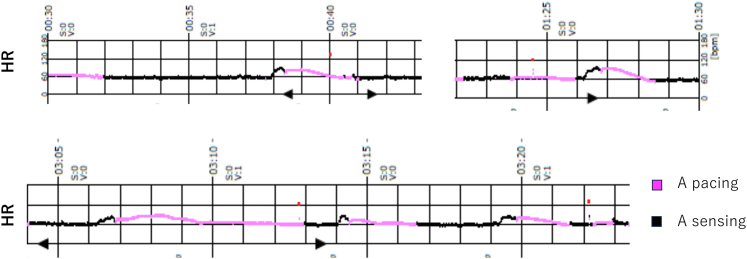
Holter monitoring reveals a cycle of elevated heart rate followed by activated closed-loop stimulation 1–2 times per hour.

Following the elevated HR, the atrial pacing was activated. As a result of atrial pacing induced by the activation of CLS, a stabilization in HR was observed without any decrease (Figure 2B). The HUT result was negative because no decrease in blood pressure or symptoms was observed. Transiently elevated HR followed by atrial pacing, similar to that observed on the HUT, was observed every night after implantation; however, there were no episodes of incontinence or feeling unwell upon waking. Holter monitoring revealed a cycle of elevated HR followed by activated CLS at 1–2 times per hour (Figure 3). The patient was discharged on the seventh day after implantation. During the 12-month follow-up, the patient did not have syncope, incontinence, or feeling of being unwell during sleep. The pacing percentage at 12 months was 32% in the atrium and 1% in the ventricle.

## Discussion

This report presents the following salient findings. First, CLS improved sleep syncope associated with VVS. Second, detailed pacing activity state during CLS treatment for VVS was noted. Sleep syncope is rare; however, several cases have been reported.^1^^,^^4^^,^^5^ Raj and colleagues^1^ reported that sleep syncope is considered a rare subtype of VVS. Approximately 96.5% of patients with sleep syncope have daytime VVS; conversely, 2.5%–21.3% of those with VVS have sleep syncope. Moreover, HUT tests are positive in 90.9% of patients with sleep syncope. The average age of patients is 46.9 years, and a significant majority, 67.6%, also present with phobias. The mechanism leading to syncope is unclear; however, syncope often occurs following gastrointestinal symptoms after waking up and is believed to be associated with hypervagotonia. More than half, 53.8%, exhibit significant bradycardia or asystole. Although reports that have documented recording of ECGs during sleep syncope are rare, Rytlewski and colleagues^6^ documented recordings from an implantable loop recorder. The rhythm initially exhibited sinus tachycardia, which then markedly slowed, followed by a junctional rhythm that also slowed, and eventually led to a 7-second period of asystole. These findings are characteristic of a cardioinhibitory type of VVS. Consistent with previous reports, the patient in this case was middle-aged. Unlike previous cases, however, she did not exhibit any phobias. Similar to past reports, she experienced daytime syncope, which typically occurred after prolonged sitting—a symptom characteristic of vasovagal syncope. Additionally, a positive HUT supports the diagnosis of VVS. Raj and colleagues categorized sleep syncope as a rare subtype of VVS, which supports our diagnosis of VVS manifesting as asystole during both prolonged sitting and sleep. Similar to the report by Rytlewski and colleagues, the ICM record in this case displayed typical findings of a cardioinhibitory type of VVS, where asystole was observed after a transiently elevated HR. Asystole during sleep was also considered as VVS owing to its appearing after a rise in HR. This case is considered a combination of true vasovagal reflex syncope and sleep syncope. One of the 3 episodes during sleep was accompanied by gastrointestinal symptoms typical of syncope. Incontinence was observed in 1 episode, which, to the best of our knowledge, has not been reported in previous cases of sleep syncope. Raj and colleagues^1^ reported only 2 cases of pacemaker therapy for sleep syncope; however, the efficacy of pacemaker therapy for sleep syncope remains unknown. In the present case, the patient had recurrent VVS, even during the day. According to the European Society of Cardiology guidelines and Japanese Circulation Society guidelines, dual-chamber cardiac pacing is recommended for patients over 40 years of age who experience severe, unpredictable recurrent syncope with asystolic syncope during HUT testing.^7^^,^^8^ Based on these criteria, we determined that the patient was an appropriate candidate for pacemaker implantation with CLS.

In both Japan and the United States, the inclusion of CLS does not influence the cost of pacemakers, as Japanese market prices remain unchanged regardless of CLS technology and all Biotronik pacemakers in the United States are equipped with CLS. However, in some other countries, there are variations in costs where CLS-free models are offered to more price-sensitive markets. Despite potential cost differences, the additional expense associated with CLS can be justified by its clinical benefits. This is supported by small-scale randomized open trials and double-blind studies that have shown that DDD-CLS reduces the frequency of recurrent syncope episodes and increases the time to the first syncope episode,^2^^,^^3^^,^^9^ thereby underscoring the efficacy of CLS in managing cardiac rhythm effectively. However, the mechanism and detailed pacing activity state of CLS in VVS have not been reported.

In the present case, a 12-lead ECG was continuously recorded during HUT to clarify the CLS activation state. As a result of atrial pacing induced by the activation of CLS, a stabilization in HR was observed without any decrease. Thus, CLS resulted in HR stability. The following mechanisms have been hypothesized to explain this phenomenon. The sympathetic nervous system becomes hyperactive while standing, elevating the HR. CLS sets the HR according to the heightened sympathetic nervous system activity and prevents it from dropping below that HR. Even if a vagal reflex occurs and the intrinsic HR decreases, the HR does not decrease because of the CLS of atrial pacing at the set HR. We speculate that the periodic activation of atrial pacing occurred because of periodic vagal reflexes during the HUT.

Furthermore, Holter monitoring showed cyclic atrial pacing following HR elevation at night. It has been suggested that the CLS functions appropriately in response to sympathetic nervous system activation during sleep, which is a mechanism underlying sleep syncope. We determined that CLS was effective for VVS during sleep because of improvement in sleep syncope symptoms.

To our knowledge, there have been no previous reports on the use of cardioneuroablation for sleep syncope. Sleep syncope, as documented in the literature, often co-occurs with daytime cardioinhibitory VVS, exhibiting similar electrocardiographic findings. Given these similarities, cardioneuroablation can be considered a potential treatment option for sleep syncope in future studies. This report is the first to discuss the utility of CLS for sleep syncope. As this is a single case report, studies with a larger population are necessary to validate the effect of CLS on sleep syncope.

## Conclusion

In this report, we report a case in which we diagnosed a rare condition of sleep syncope related to VVS and performed implantation of a DDD CLS pacemaker. Activated CLS prevented a drop in HR and syncope during daytime and sleep.

## Disclosures

Dr Kondo received lecture fees from Daiichi-Sankyo, Bayer, Abbott Medical Japan, Biotronik Japan, Boston Scientific, and Japan Lifeline, and research funds from Daiichi-Sankyo. The other authors have no conflicts of interest to declare.
